# Bi_2_O_2_Se-Based Monolithic Floating-Gate Nonvolatile Memory with Enhanced Charge Retention and Switching Performance

**DOI:** 10.1021/acsnano.5c14437

**Published:** 2025-11-17

**Authors:** Chi-Chun Cheng, Hsing-Chien Chien, Tai-Ting Lee, Yuen-Chih Chen, Huynh-Uyen-Phuong Nguyen, Sun-Zen Chen, Yung-Chang Lin, Kazu Suenaga, Chang-Hong Shen, Yu-Lun Chueh, Yen-Fu Lin, Mei-Yin Chou, Po-Wen Chiu

**Affiliations:** † Department of Electrical Engineering, 34881 National Tsing Hua University, Hsinchu 30013, Taiwan; ‡ College of Semiconductor Research, National Tsing Hua University, Hsinchu 30013, Taiwan; § 71556 Institute of Atomic and Molecular Sciences, Academia Sinica, Taipei 10617, Taiwan; ∥ Department of Physics, National Taiwan University, Taipei 10617, Taiwan; ⊥ Center for Nanotechnology, Materials Science and Microsystems, National Tsing Hua University, Hsinchu 30013, Taiwan; # Research Institute of Core Technology for Materials Innovation, National Institute of Advanced Industrial Science and Technology (AIST), Tsukuba 305-8565, Japan; ∇ The Institute of Scientific and Industrial Research (SANKEN), The University of Osaka, Osaka 567-0047, Japan; ○ Department of Materials Science and Engineering, National Tsing Hua University, Hsinchu 30013, Taiwan; ◆ Department of Physics, 34916 National Chung Hsing University, Taichung 40227, Taiwan

**Keywords:** Bi_2_O_2_Se, monolithic nonvolatile memory, low-power device, 2D memory, process simplification

## Abstract

The continuous scaling of conventional floating-gate memories faces major challenges due to charge leakage and complex multilayer architectures. Here, we report a monolithic nonvolatile memory (NVM) device constructed using a single 2D material, Bi_2_O_2_Se, that integrates channel, charge storage, and tunneling functions within the same material system. Upon UV–ozone treatment, semiconducting Bi_2_O_2_Se (s-BOS) forms a conformal and crystalline β-Bi_2_SeO_5_ shell. Subsequent thermal annealing introduces selenium vacancies into the core, converting it to metallic Bi_2_O_2_Se (m-BOS), which serves as a floating-gate capable of efficient charge trapping, while the crystalline BOS oxide shell provides robust tunneling insulation and suppresses leakage. This monolithic structure integrates channel (s-BOS), storage (m-BOS), and tunneling functions (BOS oxide) within a single material system. The devices exhibit a large memory window, a high charge storage density (∼5 × 10^13^ cm^–2^), and a current ON/OFF ratio exceeding 10^8^. They also show fast programming/erasing with ±12 V, 100 ms pulses, robust endurance over 2000 cycles, and charge retention exceeding 10^4^ seconds. Compared with other 2D NVMs employing separate materials for each functional layer, this single-material platform enables simplified fabrication and improved scalability in the 2D memory device design.

## Graphical abstract

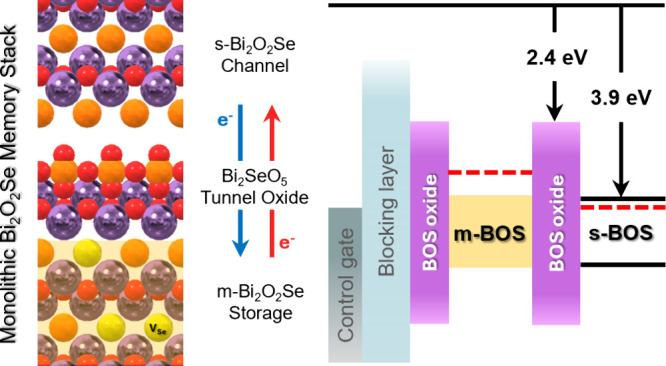

## Introduction

The explosive growth of artificial intelligence, edge computing, and the Internet of Things has fueled an increasing demand for high-performance nonvolatile memory (NVM) technologies.^1–4^ NVM devices are critical to modern electronics as they retain stored information without a continuous power supply.^5,6^ Among commercially available NVM types, floating-gate flash memory remains dominant due to its cost-effectiveness and mature integration with CMOS platforms.^7^ However, the structure of flash memories faces critical challenges,^8^ such as high programming voltages, limited endurance, and charge leakage through defective tunneling oxides,^9–11^ as devices scale down into the nanometer regime. These problems originate from the polycrystalline silicon-based floating-gates and become more pronounced at smaller dimensions, leading to reduced memory windows and compromised data retention.^12–14^ Such bottlenecks highlight the urgent demand for alternative memory designs that can offer long-term stability, scalability, and low-power operation.

To address these challenges, in addition to the state-of-the-art TANOS structure,^15–17^ a variety of emerging nonvolatile memory (NVM) alternatives have been developed,^6,18,19^ including resistive random-access memory (RRAM),^20^ phase-change memory (PCM),^21^ spin-transfer torque magnetic RAM (STT-MRAM),^22^ and ferroelectric RAM (FeRAM).^23^ Each type has distinct advantages: RRAM and PCM support fast switching and good scalability,^8,24,25^ while FeRAM and STT-MRAM offer excellent endurance and low power consumption.^6^ Nonetheless, these approaches often struggle with stochastic switching behavior, integration complexity, or limited retention time.^26,27^ Recently, two-dimensional (2D) materials have emerged as promising building blocks for next-generation NVM,^28^ owing to their atomically thin body, clean interfaces, and applicable electronic properties.^29,30^ Devices constructed from van der Waals (vdW) heterostructures—comprising graphene, transition metal dichalcogenides (TMDs), and hexagonal boron nitride (hBN)—have demonstrated promising characteristics, including high switching ratios, strong electrostatic modulation, and multiyear retention.^28^ In the conventional device structure, a vdW memory stack includes a channel, tunneling dielectric, charge-trapping layer, and a blocking layer. For example, graphene may serve as a floating-gate due to its tunable work function, while h-BN acts as a high-quality tunneling layer.^31–36^ However, despite their functional versatility, these multicomponent heterostructures suffer from poor large-scale reproducibility, potential interface variability, and fabrication complexity, which pose challenges for large-scale integration and practical device implementation.

Given the limitations of both conventional flash and stacked 2D heterostructures, the development of monolithic material systems that simultaneously support charge transport, storage, and insulation has become an important direction. In this work, we introduce a new floating-gate memory architecture based on a core–shell heterostructure^37^ derived from Bi_2_O_2_Se (Figure 1). This layered semiconductor Bi_2_O_2_Se, denoted as s-BOS, undergoes surface oxidation under UV–ozone exposure to yield a conformal, crystalline β-Bi_2_SeO_5_ (BOS oxide) shell.^38^ The resulting heterostructure with s-BOS surrounded by BOS oxide integrates the functions of a charge-trapping layer and tunneling dielectric into a single self-assembled structure, eliminating the need for separate vdW stacking. Moreover, postoxidation annealing generates selenium vacancies within the s-BOS core. These vacancies act through a self-modulation doping effect, effectively driving the transformation toward metallic-BOS (m-BOS).^39,40^ This process enhances the charge-storage capability by elevating the Fermi level closer to the conduction band. This monolithic configuration not only simplifies fabrication but also minimizes interfacial defects and trap states in the bandgap, resulting in a wide memory window, high program/erase ratios, and stable long-term retention. Our results position the BOS-based core–shell heterostructure as a potential platform for scalable, low-power, and reliable nonvolatile memory in future data-driven electronics.

**Figure 1:**
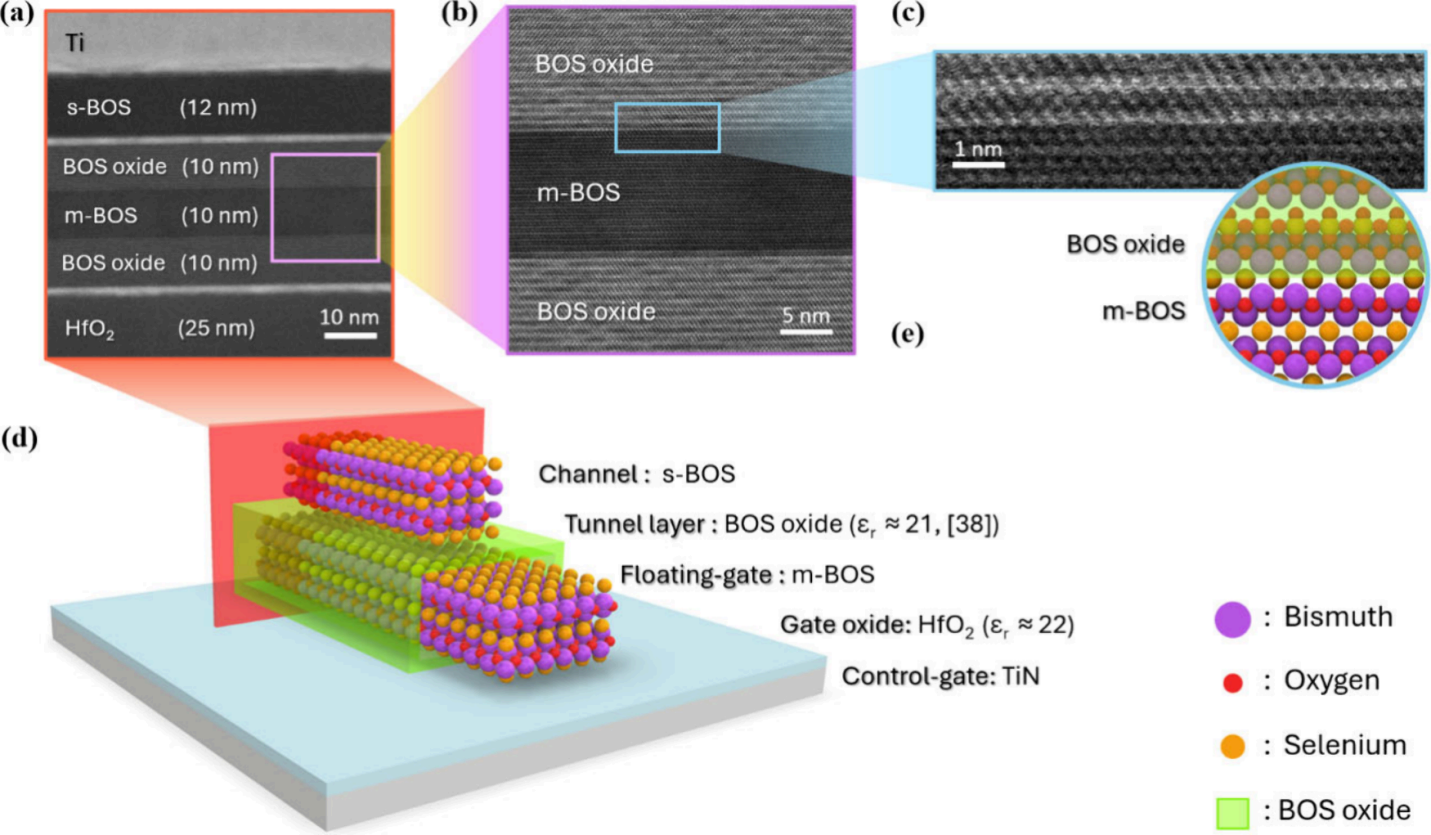
Schematic illustration and cross-sectional characterization of the BOS-based floating-gate nonvolatile memory device. Cross-sectional STEM images reveal a crystalline BOS oxide shell encapsulating the s-BOS core. The integrated structure functions as channel (s-BOS), tunneling layer (BOS oxide), and floating-gate (m-BOS) within a single material platform.

## Results and Discussion

In this study, we constructed a floating-gate NVM device made entirely from a single type of layered semiconductor material. The device features a bismuth-based oxychalcogenide active channel stacked atop another, with the bottom layer undergoing conformal surface oxidation. This process results in a core–shell heterostructure, where the s-BOS core is encapsulated by a crystalline BOS oxide shell. This self-assembled architecture enables the seamless integration of both charge storage and tunneling functionalities within a unified material system. Cross-sectional high-angle annular dark-field scanning transmission electron microscopy (HAADF-STEM) images (Figure 1) reveal the layered growth of the BOS oxide on the s-BOS surface, confirming the crystalline nature of the oxide and its epitaxial alignment.

To prepare the bottom BOS layer for use as a floating gate, thermal annealing was further employed to introduce selenium vacancies, thereby increasing the electron concentration and transforming the material into a degenerate semiconductor (m-BOS). This process enhances the charge-trapping capability of the BOS core. Figure 2a shows the transfer characteristics of a back-gated s-BOS field-effect transistor (FET) after annealing at different temperatures. As the annealing temperature increases stepwise from 25 to 300 °C, the drain current progressively rises and the threshold voltage shifts negatively. These trends indicate a systematic increase in the electron carrier concentration within the bottom BOS layer, consistent with n-type doping driven by selenium vacancy formation. At annealing temperatures above 250 °C, the devices exhibit near-metallic behavior, characterized by high off-state currents and reduced gate modulation—typical signatures of degenerate doping.

**Figure 2:**
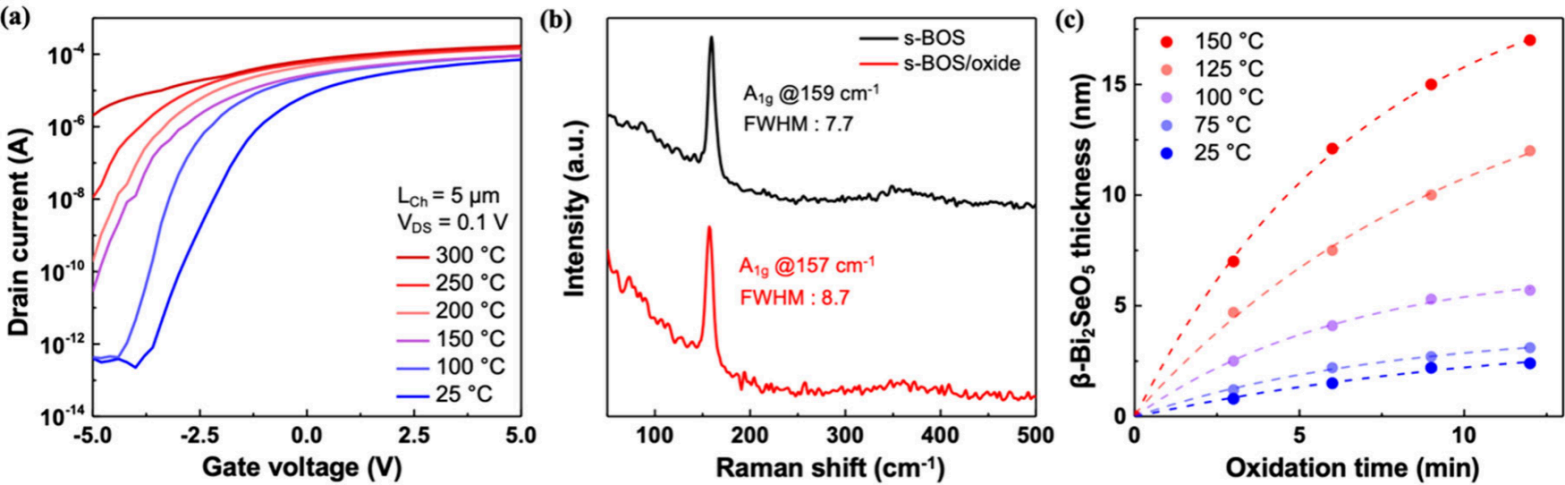
Electronic, vibrational, and structural characterization of the s-BOS/oxide heterostructure. (a) Transfer curves of s-BOS FETs annealed at different temperatures, indicating Se vacancy-induced n-type doping. (b) Raman spectra reveal a redshift and broadening of the A_1g_ mode after oxidation. (c) BOS oxide thickness as a function of oxidation time and temperature, showing thermally activated growth. The approach for thickness measurement is stated in Supporting Information 5.

To confirm the structural transformation induced by UV–ozone oxidation, Raman spectroscopy was performed on both pristine s-BOS and the resulting s-BOS/oxide core–shell structure. As shown in Figure 2b, the pristine s-BOS exhibits a sharp A_1g_ vibrational mode centered at 159 cm^–1^ with a full width at half-maximum (fwhm) of 7.7 cm^–1^, indicative of its high crystallinity and well-ordered lattice structure. Following oxidation, the A_1g_ peak undergoes a slight redshift to 157 cm^–1^ and a noticeable broadening to a fwhm of 8.7 cm^–1^. The redshift reflects phonon softening, which may arise from altered interlayer interactions or lattice relaxation induced by the formation of the crystalline BOS oxide. Importantly, high-resolution STEM imaging also confirms the long-range order and crystallinity of the BOS oxide shell, ruling out amorphous disorder as the primary cause. These observations support the formation of a structurally coherent core–shell architecture with modified vibrational characteristics near the surface.

Precise control over the thickness of the BOS oxide shell is critical for achieving optimal tunneling characteristics and device reliability. To calibrate the oxidation kinetics, we employed lithographic patterning and selective wet etching followed by atomic force microscopy (AFM) to extract the oxide thickness as a function of oxidation temperature and time. Figure 2c illustrates the growth of the β-Bi_2_SeO_5_ surface oxide as a function of the oxidation time and temperature. The BOS oxide thickness exhibits a nonlinear increase with oxidation duration, and the growth rate is strongly temperature-dependent. At 125 °C, the oxide layer grows rapidly, reaching ∼10 nm within 8 min. In contrast, significantly thinner shells are obtained at lower temperatures, with sub 5 nm thicknesses even after extended oxidation at 75 °C or below. The observed saturation behavior suggests a diffusion-limited growth mechanism. The oxide thickness increases systematically with both oxidation time and temperature, showing the controllability of the UV–ozone oxidation process. For all device fabrication in this study, an oxidation condition of 125 °C for 8 min was selected to ensure a consistent ∼10 nm tunneling barrier that balances charge confinement and efficient Fowler–Nordheim tunneling.

Density functional theory (DFT) calculations further support the experimentally observed n-type doping behavior induced by thermal annealing. As shown in Figure 3, the calculated density of states (DOS) of the m-BOS model containing ∼3.1% selenium vacancies shows that the Fermi level is shifted into the conduction band without introducing midgap states, indicating a transition toward metallic behavior. This aligns well with our electrical measurements (Figure 2a), which show a monotonic increase in channel conductivity and a suppressed gate modulation with an increasing annealing temperature. Using a hybrid exchange-correlation functional, the calculated band gap of s-BOS is 1.15 eV, while β-Bi_2_SeO_5_ has a wide bandgap of 3.70 eV, similar to that of the other oxide phase, α-Bi_2_SeO_5_. We adopted the work functions of Bi_2_O_2_Se and α-Bi_2_SeO_5_ measured by ultraviolet photoelectron spectroscopy^38^ to align the band energies between s-BOS and the BOS oxide. The resulting large valence and conduction band offsets are 1.10 and 1.45 eV, respectively, as shown in Figure 3c. As a result, the Fermi level of m-BOS resides deep within the energy gap of the BOS oxide. Together, these theoretical and experimental findings confirm that controlled generation of Se vacancies enables electronic structure tuning in s-BOS, transforming it into a degenerate n-type semiconductor that is well-suited for charge storage in nonvolatile memory applications.

**Figure 3:**
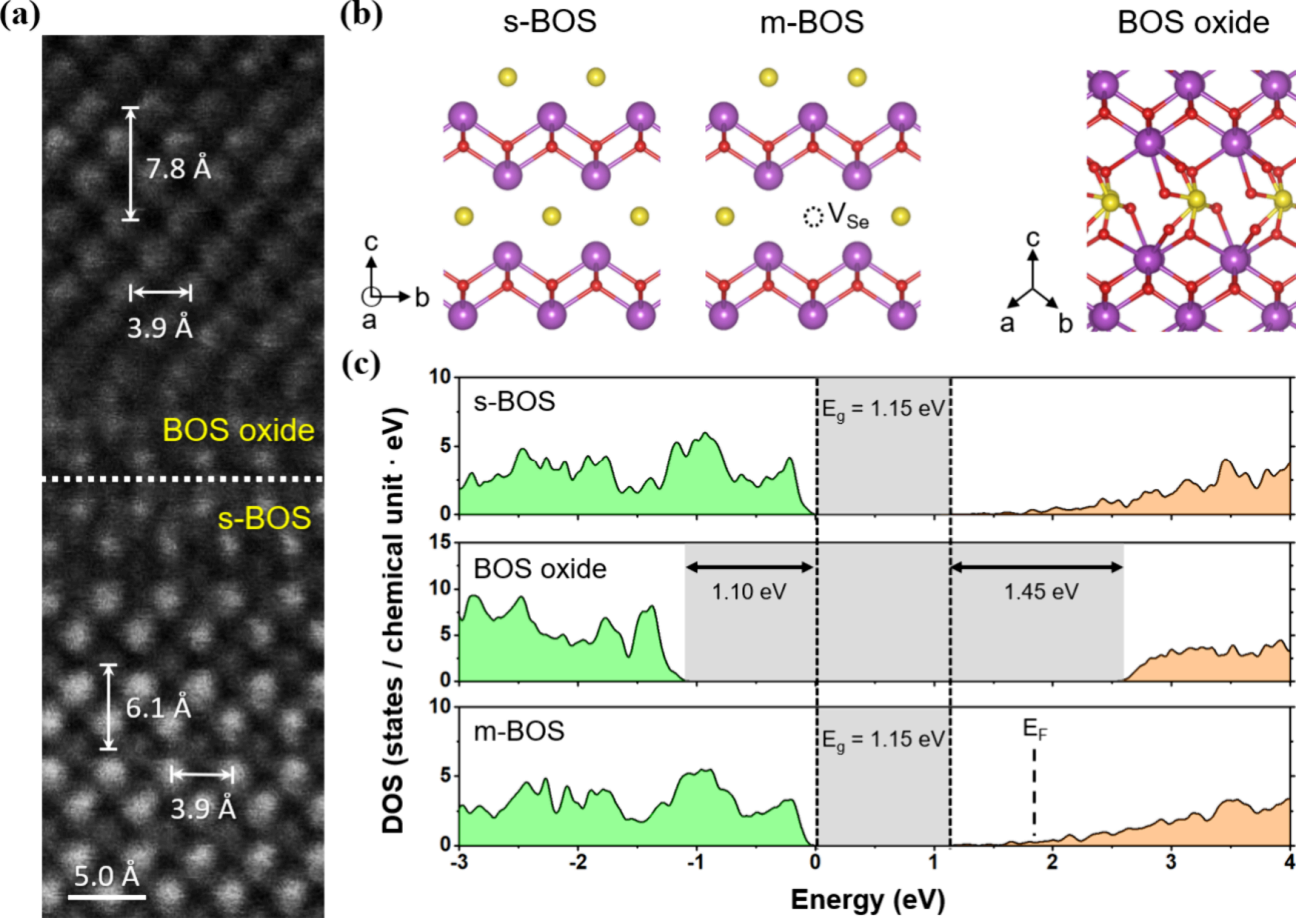
STEM characterization of the s-BOS/oxide heterostructure and DFT calculations. (a) Atomic-resolution STEM image showing a BOS oxide atop s-BOS. (b) Atomic structures of pristine s-BOS, m-BOS with selenium vacancies, and the native BOS oxide. The purple, red, and yellow atoms represent Bi, O, and Se, respectively. (c) Density of states (DOS) for s-BOS, the BOS oxide, and m-BOS with 3.1% selenium vacancies. Band gap regions are shaded in gray. The Fermi level of m-BOS is marked by a black dashed line, showing an n-type degenerate semiconductor. The valence band maximum of s-BOS is set to energy zero, while the band energies of the BOS oxide and s-BOS are aligned based on their experimentally measured work functions.^38^

The interface transformation from semiconducting Bi_2_O_2_Se to metallic Bi_2_O_2_Se primarily occurs through selenium vacancy formation rather than large-scale phase boundary migration. During UV–ozone oxidation, oxygen atoms react with selenium near the surface, forming a conformal crystalline β-Bi_2_SeO_5_ shell. Subsequent thermal annealing promotes localized Se vacancy generation within the core, as evidenced by the systematic increase in carrier concentration (Figure 2a) and supported by DFT calculations (Figure 3c), which show that Se vacancies raise the Fermi level into the conduction band without introducing midgap states. High-resolution STEM images further confirm the structural integrity of the core–shell interface, ruling out a sweeping, zipper-like transformation. This vacancy-driven process enables a gradual and controlled transition to an m-BOS core surrounded by a stable BOS oxide shell, which is critical for reliable memory operation.

To evaluate baseline electronic performance, we further fabricated s-BOS FETs with Ti/Au source/drain contacts and HfO_2_ gate oxide (25 nm) deposited via plasma-enhanced atomic layer deposition (Figure 4a).^38^ These FETs exhibited excellent n-type behavior, including an on/off current ratio exceeding 10^7^ and a subthreshold swing (SS) of ∼360 mV/dec (Figure 4b). Output characteristics confirmed ohmic contact with a low output resistance (∼800 Ω) at V_BG_ = 9 V.

**Figure 4:**
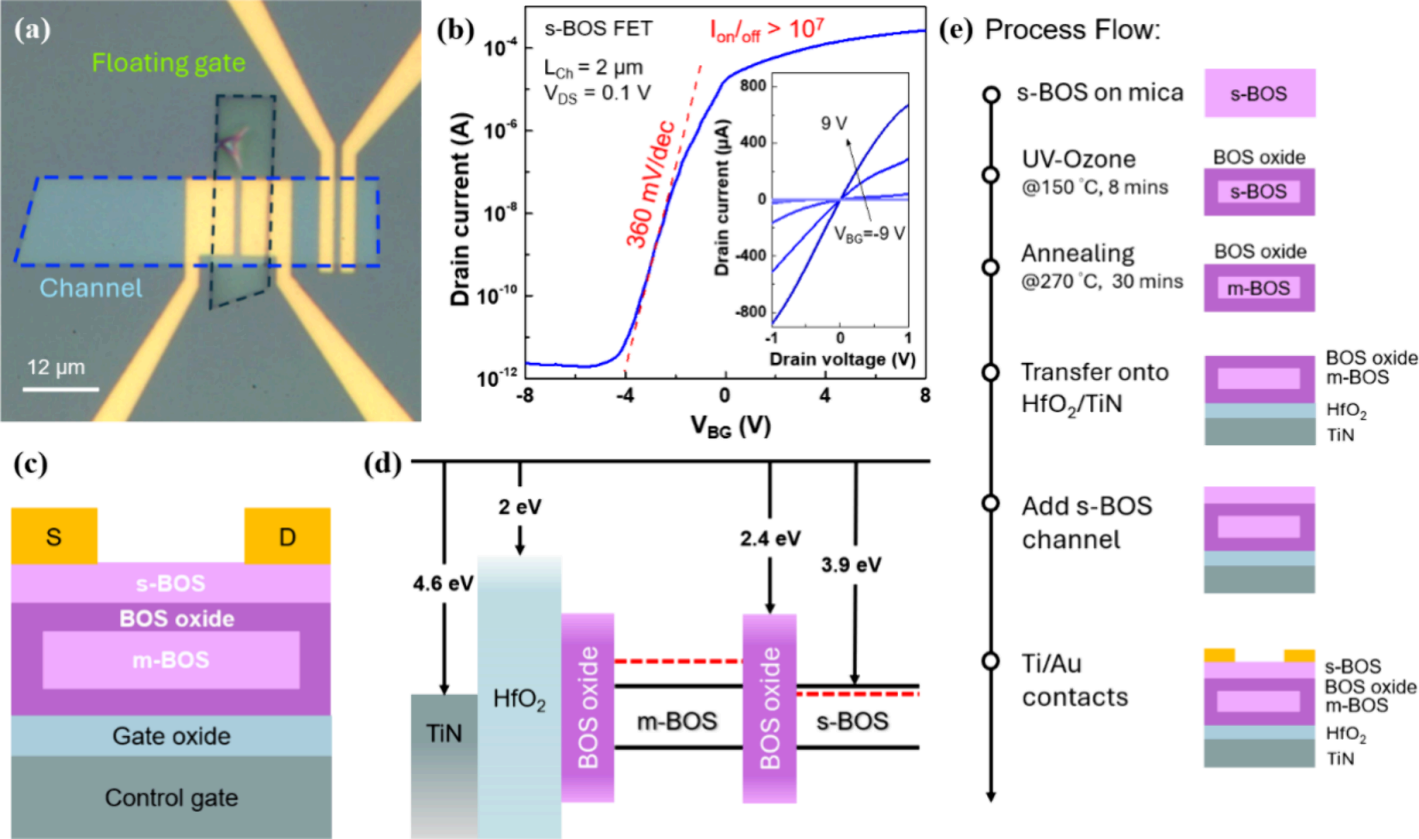
Electronic properties of the s-BOS FET and BOS NVM structure. (a) Optical image of a BOS NVM device arranged in a cross structure. A control device of s-BOS back-gate FET is fabricated on the same s-BOS crystal, located in the right-hand side of the BOS NVM device. (b) Transfer characteristics of the back-gate FET, with inset showing linear I–V output curves. (c) Schematic of the BOS NVM device structure incorporating the BOS floating-gate stack above a HfO_2_/TiN substrate. (d) Energy band diagram and electron affinity of the corresponding layers. The bandgap values are as follows: BOS, 1.15 eV; BOS oxide, 3.7 eV; HfO_2_ (grown by PE-ALD), ∼5.8 eV.^41^ (e) Process flow for the whole BOS NVM device. Two pieces of s-BOS on mica are needed; one is for the m-BOS/oxide core–shell structure and the other is for the channel.

By integrating the m-BOS/oxide core–shell structure between the channel and the gate stack, we constructed a prototype floating-gate NVM device, as schematically shown in Figure 4c. The energy band alignment, depicted in Figure 4d, shows that the BOS oxide tunneling layer, with a bandgap of ∼3.7 eV and electron affinity of 2.4 eV, forms an effective barrier of ∼1.5 eV relative to the m-BOS core. Resulting floating-gate structure enables efficient charge confinement and tunneling injection.

Electrical measurements of the BOS-based floating-gate memory device under varying gate voltage sweeps demonstrated robust nonvolatile memory characteristics. As shown in Figure 5a, a pronounced hysteresis is observed in the transfer curves for increasing control-gate voltage ranges (±6, ±9, and ±12 V), indicating effective charge storage and release within the core–shell floating gate. The memory window, defined by the threshold voltage difference between forward and reverse sweeps, increases nonlinearly with the sweep voltage, reaching approximately 12 V under a ±12 V control-gate sweep (Figure 5a, inset). This represents a utilization of nearly 50% of the applied voltage span, comparable to or higher than reported 2D van der Waals (vdW) NVMs.^28^ The subthreshold swing remains below 400 mV/dec, and the program/erase current ratio (I_E_/I_P_) exceeds 10^8^, reflecting excellent switching contrast and gate control. Based on the HfO_2_ gate dielectric thickness (25 nm, ε_r_ ≈ 22), the stored charge density in the floating gate was estimated to be ∼5 × 10^13^cm^–2^, showing higher charge storage density compared with conventional vdW memory platforms employing graphene or TMDs.^32^

**Figure 5:**
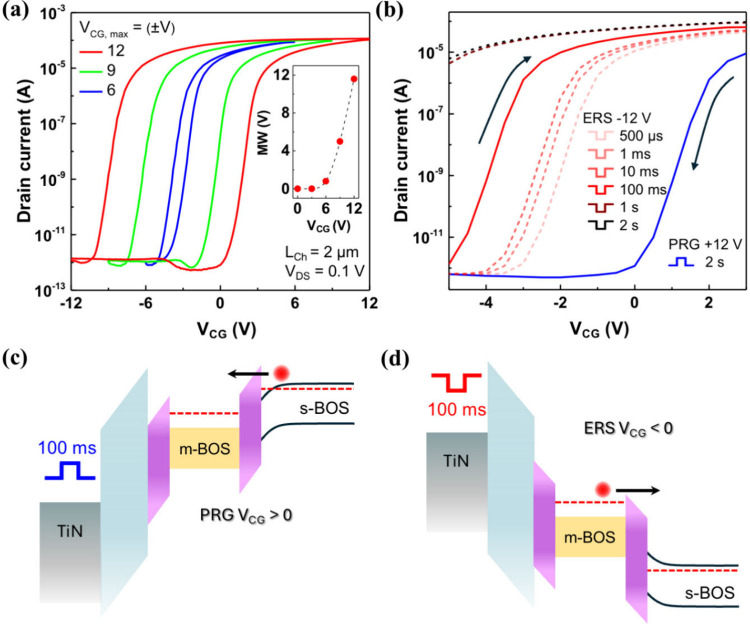
Memory switching characteristics and charge transfer mechanisms of the BOS NVM device. (a) Transfer curves under increasing control gate voltage V_CG_ (±6 to ±12 V), showing widening memory window. Inset: memory window versus gate sweep amplitude. (b) Dynamic switching behavior with fixed PRG pulse (+12 V, 2 s) and varying ERS pulse durations (−12 V, 0.5 ms to 2 s), illustrating progressive threshold voltage recovery. (c, d) Schematic energy band diagrams depicting charge injection during programming (c) and erasing (d) via Fowler–Nordheim and field-assisted tunneling through the BOS oxide barrier.

We next assessed the dynamic program/erase (PRG/ERS) characteristics of the BOS-based floating-gate memory by applying ±12 V control-gate voltage pulses of varying durations. Figure 5b presents the dynamic transfer characteristics of the device under programmed (+12 V for 2 s) and erased (−12 V) conditions with varying erase pulse durations ranging from 500 μs to 2 s. The blue curve corresponds to the programmed state, showing a significant positive shift in threshold voltage due to trapped electrons in the floating gate. Following this, a series of erase pulses with increasing duration (from pink to dark red) were applied to evaluate the speed and completeness of the charge removal. The results reveal a strong dependence of erase efficiency on the pulse duration. For short ERS pulses (≤1 ms), the threshold voltage shift is minimal, indicating incomplete charge extraction. As the duration increases to 10 and 100 ms, the transfer curves shift progressively toward the unprogrammed state. At 1 and 2 s, the device fully recovers to a low-threshold state, effectively erasing the stored charge and restoring the initial transfer characteristics. This gradual transition indicates a time-dependent tunneling mechanism governed by the electric field across the BOS oxide tunneling barrier. The subthreshold region of each curve remains steep, suggesting that gate control is maintained, even during dynamic operations. This performance highlights the efficiency of field-assisted electron emission from the m-BOS layer through the crystalline tunneling BOS oxide.

Figures 5c and 5d schematically illustrate the charge transport mechanisms during the PRG and ERS modes, respectively. Under positive control-gate bias (PRG), electrons tunnel from s-BOS through the BOS oxide barrier into the m-BOS (n^+2^) floating-gate via Fowler–Nordheim tunneling. Conversely, during ERS operation (negative V_CG_), the stored electrons are extracted from the floating gate back into the s-BOS via field-assisted tunneling. These results confirm efficient bidirectional charge transfer and underline the suitability of the core–shell m-BOS/oxide floating gate for fast, low-voltage, nonvolatile memory operations.

The endurance and retention performance of the BOS NVMs were evaluated to assess their long-term reliability. As shown in Figure 6a, the device underwent 2000 program/erase (PRG/ERS) cycles using ±12 V pulses (100 ms duration each), with the drain current (I_D_) monitored at a constant read bias of V_DS_ = 0.1 V and V_CG_ = −2 V. Throughout the cycling test, the device consistently exhibited well-separated high (ERS) and low (PRG) current states, with an I_on_/I_off_ ratio exceeding 10^7^ and no significant degradation, demonstrating excellent endurance and repeatability.

**Figure 6:**
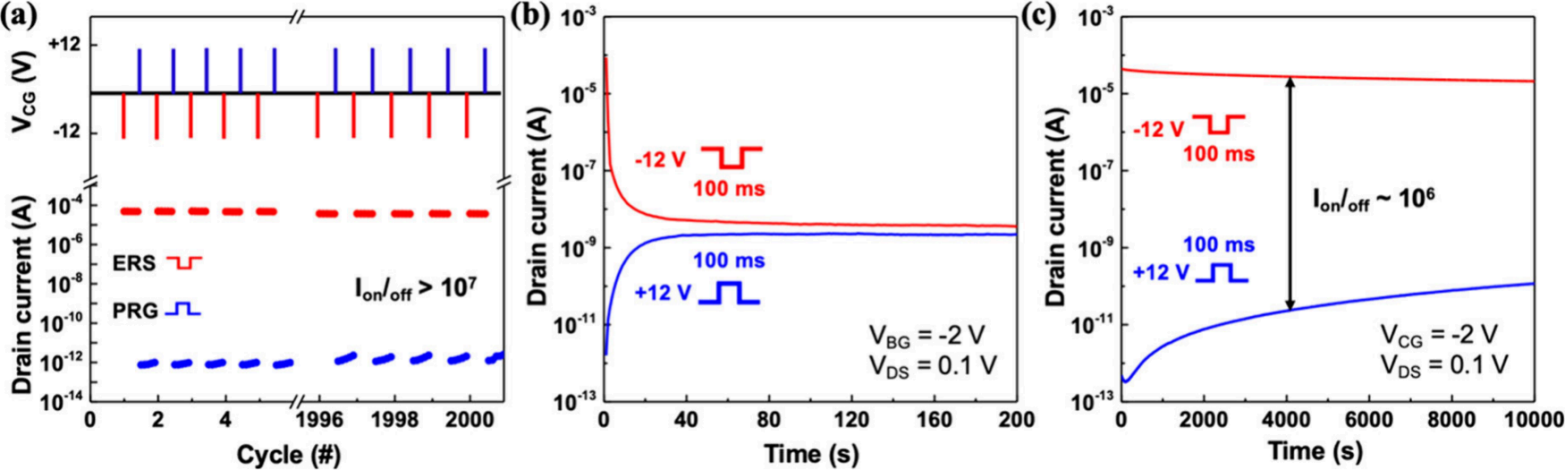
Endurance and retention performance of the BOS NVM device. (a) Endurance test over 2000 PRG/ERS cycles (±12 V, 100 ms). (b) Retention characteristics of a control device without the BOS oxide shell showing rapid current degradation within 200 s. (c) Retention test of the device demonstrating stable I_on_/I_off_ ≈ 10^6^ over 10^4^ s.

Figures 6b and 6c compare the retention performance of memory devices without and with the BOS oxide tunneling shell, respectively. In Figure 6b, the control device lacking a well-defined floating gate structure shows rapid degradation of both program and erase states, with the I_on_/I_off_ ratio collapsing within 100 s. This reflects severe charge leakage, likely due to insufficient tunneling insulation. In contrast, the device with BOS oxide as a tunnel barrier (Figure 6c) retains stable high and low current states for over 10^4^ s after ±12 V, 100 ms PRG/ERS pulses. The current levels remain well separated, with an I_on_/I_off_ ratio on the order of 1 × 10^6^. This performance demonstrates the critical role of the crystalline shell in suppressing leakage and maintaining robust nonvolatility. Also, the intrinsic stability of our BOS NVM system is supported by the stable retention and endurance, indicating that the structure remains electrically and mechanically robust even under repeated stress. No significant threshold voltage drift or leakage pathways were observed during these tests, which is a key indicator of stability for memory devices.

Finally, we benchmarked the performance of the BOS NVM devices against representative 2D vdW memory systems reported in the literature, including devices based on MoS_2_, WS_2_, ReS_2_, and WSe_2_/WS_2_ heterostructures (Table 1). The BOS NVM device exhibits competitive performance across multiple key metrics. It exhibits a memory window of 50% and a high charge storage density of 5.5 × 10^13^ cm^–2^—higher than those of other 2D counterparts, which typically range from 0.1 to 3 × 10^12^ cm^–2^. The I_E_/I_P_ current ratio reaches 10^8^, which is orders of magnitude higher than those of MoS_2_ (∼10^4^), WS_2_ (∼10^3^), and ReS_2_ (∼10^4^) memories. In terms of dynamic performance, the BOS NVM achieves these characteristics using moderate programming/erasing pulses (±12 V, 100 ms), surpassing devices requiring much longer pulse widths but with lower retention fidelity. Importantly, the endurance of our BOS NVM devices exceeds 2000 cycles, and it maintains charge retention for over 10^4^ s, matching ReS_2_ and MoS_2_ memories that exhibit a similar or shorter retention time. These comparisons highlight the BOS NVM architecture as a high-performance and scalable memory platform that unifies large charge capacity, robust switching contrast, fast operation, and long-term stability.

**Table 1:** Benchmark of BOS NVM Device against Other NVM Technology, Including 2D Materials,^32–36^ SONOS (Silicon-Oxide-Nitride-Oxide-Silicon),^42^ and TANOS (TaN-Al_2_O_3_-Nitride-Oxide-Silicon)^43^

device type/stack		Vop	MW (ΔVth)	charge density (10^12^/cm^2^)	I_E_/I_P_	endurance	retention	reference
Bi_2_O_2_Se monolithic structure		+12 V/–12 V, 100 ms pulse	12 V	55	10^8^	>2000 cycles	>10^4^ s	this work
TiN/HfO_2_/m-BOS/BOS oxide/s-BOS								
charge layer/tunneling layer	WSe_2_WS_2_	+80 V/–80 V, 100 ms pulse	34 V	2.5	10^6^	>1000 cycles	93% in 10 years	^32^
	WS_2_	+20 V/–20 V, 100 ms pulse	∼5 V	0.85	10^3^	-	87% in 10 years	^33^
	ReS_2_	+30 V/–30 V, 10 ms pulse	38 V	2.9	10^4^	>1000 cycles	>10^4^ s	^34^
graphene/h-BN	MoS_2_	+40 V/–40 V, 1 ms pulse	20 V	0.1∼10	10^4^	>100 cycles	>10^3^ s	^35^
	MoS_2_	+35 V/–35 V, 50 ps pulse	53 V	0.54	10^6^	1390 cycles	10 years	^36^
BE-SON(ONO)S, polySi/SiO_2_/Si_3_N_4_(SiO_2_/Si_3_N_4_/SiO_2_)		+19 V/–18 V, 10 ms pulse	∼8.0 V	-	-	>10^4^ cycles	81% in 10 years	^42^
BE-MON(AHO)S, TiN/SiO_2_/Si_3_N_4_(AILaO_3_/HfAlO/SiO_2_)			∼14.9 V	-	-	>10^4^ cycles	87% in 10 years	
TANOS, TaN/Al_2_O_3_/Si_3_N_4_/SiO_2_		+18 V, 10 ps/–24 V, 2 ms	∼3 V	-	-	>10^4^ cycles	63% in 10 years	^43^

We also compare the conventional SONOS and CTF­(SiN)-based TANOS flash memory which relies on a complex multilayer stack consisting of a low-κ SiO_2_ tunneling oxide (∼3.9), a Si_3_N_4_ charge-trap layer (∼7.5), and a high-κ Al_2_O_3_ blocking layer (∼9).^42,43^ This structure enables capacitance coupling, concentrating most of the applied program voltage across the thin SiO_2_ tunneling layer to generate a strong electric field for efficient Fowler–Nordheim tunneling. While TANOS remains the dominant commercial technology, it faces scaling limitations due to interfacial defects, charge leakage, and process variability arising from multiple deposition and etching steps. In contrast, the Bi_2_O_2_Se monolithic device reported here integrates all three functional layers within a single crystalline material system. The β-Bi_2_SeO_5_ shell forms naturally through a self-limiting oxidation process, eliminating the need for separate materials and interfaces. This results in a structurally coherent tunneling barrier with low defect density, improving charge retention and reliability. Furthermore, the simplified fabrication flow of Bi_2_O_2_Se devices can enhance process efficiency and yield as it avoids the lithographic complexity of conventional TANOS stacks. From a performance standpoint, our prototype demonstrates a large memory window of 12 V at ±12 V sweep, ultrahigh program/erase ratio (>10^8^), and stable endurance over 2000 cycles, which is comparable to early generation TANOS devices. While additional engineering is required to meet full industrial reliability standards, these results highlight the potential of Bi_2_O_2_Se as a scalable, low-interface, and manufacturing-friendly platform for future nonvolatile memory technologies.

## Conclusions

This study demonstrates a monolithic design concept for nonvolatile memory, showing that all essential device functions, including channel transport, charge storage, and tunneling insulation, can be realized within a single 2D material system. Through surface oxidation of Bi_2_O_2_Se, a crystalline β-Bi_2_SeO_5_ shell forms in situ, serving as a robust tunneling dielectric while preserving the underlying layered framework. Subsequent defect engineering via thermal annealing transforms the semiconducting core into a metallic state, enabling efficient charge confinement without the introduction of additional interfaces. By unifying multiple roles into a monolithic core–shell heterostructure, this approach reduces the complexity and variability associated with multimaterial van der Waals stacking, while maintaining precise control over electronic properties through intrinsic structural transformations. The resulting platform offers a scalable, CMOS-compatible pathway toward high-density, low-power, and interface-stable NVM technologies.

## Methods

### Growth of Free-Standing Bi_2_O_2_Se

Bi_2_O_2_Se (s-BOS) crystals were synthesized via low-pressure chemical vapor deposition on a fluorophlogopite mica (f-mica) substrate. A mixture of Bi_2_Se_3_ and Bi_2_O_3_ powders (99.99% purity) was arranged in an interleaved pattern in a quartz boat, with the f-mica substrate placed face-down above the powders. The system was evacuated to 5 × 10^–2^ Torr and backfilled with argon at 200 sccm. After moisture removal at 120 °C, the temperature was ramped to 580 °C at 400 Torr and held for 8 min.

### Formation of BOS Floating-Gate Structure

The free-standing s-BOS was oxidized using a UV–ozone system equipped with 254 and 185 nm spiral UV lamps and an oxygen inlet. Generated ozone and oxygen radicals intercalated into the Se sublayers of s-BOS, forming a crystalline BOS oxide shell while preserving the Bi_2_O_2_ framework. A UV filter was used to block sub 240 nm wavelengths and prevent structural damage. Postoxidation annealing at 270 °C in argon for 30 min introduced selenium vacancies to produce m-BOS cores.

### Material Transfer and Device Fabrication

Dry mechanical transfer was used to transfer s-BOS and m-BOS/BOS oxide core–shell structures onto target substrates. First, the mica substrate with the material was attached to a glass slide using PDMS (poly­(dimethylsiloxane)) on the backside. Using our custom transfer device, the slide was inverted with the material facing the target substrate. Since mica, PDMS, and the glass slide are all transparent, the material morphology could still be observed through the microscope during the process. The glass slide was then gently lowered by using a special holder and brought near the target substrate. When the material contacted the substrate, bending of the BOS crystal could be observed through the microscope. The height was then further adjusted, and slight horizontal movements were made. The color changes when the material detached indicated successful attachment to the substrate. The transfer details are shown in Supporting Information 2.

The NVM device undergoes two dry transfers, as shown in Figure 4e. After UV–ozone treatment, s-BOS is immediately subjected to a 270 °C treatment for 30 min, forming m-BOS/BOS oxide core–shell crystals, which are then transferred to a back-gate substrate with 25 nm HfO_2_. Next, the s-BOS crystal, which will serve as the channel, is transferred on top of the above structure in a cross-pattern. Finally, source/drain electrodes are defined using electron beam lithography, with Ti (15 nm)/Au (40 nm) as the electrode material. This cross-pattern structure ensures that the channel BOS is controlled by the floating-gate, while minimizing the contact area between the floating-gate and the source/drain, reducing leakage current.

### Material and Device Characterization

Raman spectroscopy (Thermo DXR, 523 nm laser, 10 mW) was used to monitor vibrational modes before and after oxidation. STEM imaging (FEI Talos F200X in HAADF mode) confirmed the core–shell interface structure. AFM (Bruker Dimension ICON, tapping mode) was employed to measure the oxide thickness via etch step height differences. Electrical measurements were conducted by using a Keysight B1500A semiconductor analyzer to obtain transfer/output curves, endurance cycles, and charge retention characteristics of the BOS NVM devices.

### DFT Calculations

We have performed first-principles calculations based on density functional theory as implemented in the Vienna Ab initio Simulation Package (VASP).^44^ The effects of core electrons are represented by the projector-augmented-wave (PAW) pseudopotential.^45^ The energy cutoff of the plane waves was 600 eV. The Perdew–Burke–Ernzerhof (PBE)^46^ exchange-correlation functional was used for structural optimization and electronic structure calculations. To more accurately determine the bandgap, the Heyd–Scuseria–Ernzerhof (HSE06) hybrid functional^47^ was used to calculate the energies of the valence band maximum (VBM) and conduction band minimum (CBM), from which the HSE bandgap was extracted. The bandgap of s-BOS increases from the PBE value of 0.51 to 1.15 eV (HSE06), while the bandgap of the BOS oxide increases from the PBE value of 2.72 to 3.70 eV (HSE06). Therefore, we applied scissor corrections of 0.64 and 0.98 eV to the PBE electronic density of states (DOS) of s-BOS and the BOS oxide, respectively. Spin–orbit coupling was not included in these calculations. For structural relaxation, we used the experimental lattice constants and performed internal atomic relaxation until the residual force on each atom was less than 0.01 eV/Å. The lattice parameters were set as *a* = *b* = 3.88 Å, *c* = 12.16 Å for s-BOS and *a* = *b* = $\sqrt{2}$ × 3.88 = 5.49 Å, *c* = 15.60 Å for β-Bi_2_SeO_5_. The enlarged unit cell for the BOS oxide was used to explore various O arrangements, and the in-plane lattice matching was adopted following the observation of a conformal interface in the image data in Figure 3a. A Monkhorst–Pack k-point mesh of 9 × 9 × 3 was used for s-BOS, and 6 × 6 × 2 was used for the BOS oxide. The m-BOS structure was modeled by removing two Se atoms from a 4 × 4 × 2 supercell of bulk s-BOS, yielding a V_Se_ concentration of 3.1%. For this supercell, a Monkhorst–Pack k-point mesh of 2 × 2 × 2 was employed.

## Supplementary Material



## Notes

Additional experimental details and figures, including the low-pressure chemical vapor deposition (LPCVD) setup for Bi_2_O_2_Se growth, the polymer-free dry transfer process, the UV–ozone oxidation mechanism, STEM–EELS analysis of the s-BOS/oxide heterostructure, and AFM-assisted oxide thickness measurements (PDF)
