# Supplementary material for: Bi2O2Se-Based Monolithic Floating-Gate Nonvolatile Memory with Enhanced Charge Retention and Switching Performance
Source: ACS Nano. 2025 Nov 17;19(47):40497–505. doi: 10.1021/acsnano.5c14437 (PMC12676734; doi:10.1021/acsnano.5c14437)
Supplement: Supplementary file 1 [file nn5c14437_si_001.pdf]

Bi_2O_2Se-Based Monolithic Floating-Gate Nonvolatile Memory with Enhanced Charge Retention and Switching Performance

Chi-Chun Cheng[1], Hsing-Chien Chien[2], Tai-Ting Lee[3,4], Yuen-Chih Chen[1], Huynh-Uyen-Phuong Nguyen[2], Sun-Zen Chen[5], Yung-Chang Lin[6], Kazu Suenaga[7], Chang-Hong Shen[2], Yu-Lun Chueh[8], Yen-Fu Lin[9], Mei-Yin Chou[3,4], and Po-Wen Chiu[1,3]

[1]*Department of Electrical Engineering, National Tsing Hua University, Hsinchu 30013, Taiwan.*

[2]*College of Semiconductor Research, National Tsing Hua University, Hsinchu 30013, Taiwan.*

[3]*Institute of Atomic and Molecular Sciences, Academia Sinica, Taipei 10617, Taiwan.*

[4]*Department of Physics, National Taiwan University, Taipei 10617, Taiwan.*

[5]*Center for Nanotechnology, Materials Science and Microsystems, National Tsing Hua University, Hsinchu 30013, Taiwan.*

[6]*Research Institute of Core Technology for Materials Innovation, National Institute of Advanced Industrial Science and Technology (AIST), Tsukuba 305-8565, Japan.*

[7]*The Institute of Scientific and Industrial Research (SANKEN), The University of Osaka, Osaka 567-0047, Japan.*

[8]*Department of Materials Science and Engineering, National Tsing Hua University, Hsinchu 30013, Taiwan.*

[9]*Department of Physics, National Chung Hsing University, Taichung 40227, Taiwan.*

Supplementary Details

S1. Low-pressure chemical vapor deposition system for Bi_2O_2Se crystals

Bi_2O_2Se (s-BOS) crystals were synthesized using a horizontal 1-inch quartz tube furnace via low-pressure chemical vapor deposition (LPCVD). The furnace center can achieve 1000 °C, exceeding the melting point of both precursors. Prior to the growth, the quartz tube was purged with 100 sccm of argon (Ar) for several minutes to remove residual particles and moisture.

During the growth, a mica substrate was positioned facing downward above the precursor powders to promote uniform vapor-phase deposition. Bi_2Se_3 and Bi_2O_3 powders were arranged in a chessboard pattern at the bottom of a quartz boat (Fig. S1), serving as the Se and O sources, respectively. To prevent powder displacement due to static charging, a drop of deionized (DI) water was carefully applied beside the quartz boat in the furnace before loading the boat. Subsequent heating and pressure parameters followed those described in the Method section of the main text.

S2. Polymer-free dry transfer of BOS crystals

One of the advantages of the free-standing BOS crystals grown in this study is their extremely small contact area with the mica substrate. Due to the low adhesion force, the material can be transferred onto a target substrate using natural electrostatic forces. This process is entirely polymer-free, allowing us to evaluate the transfer on pristine surfaces and minimize unnecessary interference from additional processing steps.

Before initiating the transfer process, careful selection of the target material and precise determination of the transfer position are required. This preparatory step is essential since, similar to other two-dimensional semiconductors, the thickness of BOS significantly affects its band degeneracy and thus its electronic properties. Therefore, a preliminary screening is performed to select thinner flakes that are more suitable as semiconducting channels. The detailed process flow for the polymer-free dry transfer of free-standing BOS is illustrated in Figure S2.

First, the back side of the mica substrate was attached to a glass slide using polydimethylsiloxane (PDMS). In conventional dry transfer techniques, PDMS is often used to pick up flakes or polymer films because its adhesion can be released upon heating, making it a convenient tool for material handling. However, in our process, PDMS is used solely to adhere the mica to the glass slide and can in fact be replaced with any transparent and adhesive material. The glass slide was then mounted on a height-adjustable stage and clamped in place. Under continuous observation with an optical microscope, the slide was gradually lowered to bring the BOS crystal closer to the target substrate. As the gap between the two surfaces decreased, the microscope focus was adjusted accordingly, allowing the substrate patterns to become progressively clearer (Figure S2a–c).

When the material was about to make contact, it bent toward the substrate due to attractive forces, as shown in Figure S2c. If the slide was lowered further, the thin material could fold. To avoid this, as illustrated in Figure S2d, the material was gently dragged to flatten the contact surface while the distance was reduced. Once the material was fully adhered to the substrate, its color turned white due to complete light reflection, as seen in Figure S2e.

S3. UV–ozone Oxidation

As-grown s-BOS crystals on mica exhibited a free-standing structure (Figure S2a), allowing strain relaxation during oxidation and minimizing wrinkle formation. UV–ozone oxidation was performed using a SAMCO UV-1 system equipped with a helical ultraviolet lamp (primary wavelengths: 254 nm and 185 nm) and a ring-shaped oxygen inlet to ensure uniform illumination and gas formation.

Upon flowing O_2 into the chamber, 254 nm UV light dissociated oxygen molecules into atomic oxygen, which then reacted with ambient O_2 to form ozone (O_3). Subsequent irradiation with 185 nm UV light decomposed ozone into highly reactive oxygen radicals, following the reactions:

85% (254 nm):

$O_2 \rightarrow O^* + O^*$ (Atomic oxygen)

$O_2 + O^* \rightarrow O_3$

15% (185 nm):

$O_3 \rightarrow O_2 + O^*$ (Oxygen free radical)

Oxidation was carried out at atmospheric pressure with the substrate placed on a pre-heated stage at 150 °C. Oxygen gas was introduced at a flow rate of 0.25 L/min, and after a 10 min environment stabilization, the UV lamps were switched on to initiate oxidation. A 240 nm optical filter was placed above the sample to block shorter-wavelength photons that could damage the forming BOS oxide. The resulting oxide shell exhibited the crystalline β-Bi_2SeO_5 phase with conformal coverage on the BOS surface (Figure S3c).

We also show the interface of s-BOS/oxide in Figure S4d, where the mechanism for forming β-Bi_2SeO_5 can be observed. The distinctive feature of β-Bi_2SeO_5 is that it has a lattice structure similar to that of s-BOS, with the only difference being the increased thickness due to oxygen insertion. In the figure, the oxygen atoms are shown to insert between the s-BOS layers and react with the Se layer.

S4. STEM–EELS analysis of the BOS and its oxide.

The STEM image and elemental mappings from EELS (acquired using a Thermo Fisher Scientific FEI Talos F200X operated in STEM/HAADF mode with EELS line-scan capability) clearly revealed a distinct bilayer structure, consisting of the pristine s-BOS and the BOS oxide at the bottom and top, respectively. The elemental distribution maps of Bi, O, and Se further confirmed the spatial separation of the two regions, with higher oxygen signals predominantly localized in the BOS oxide while lower signal at the s-BOS region.

The interface between them was atomically flat, indicating a coherent and well-defined boundary. Such high-quality interfaces were crucial to minimize interfacial defect density, which is often the origin of charge trapping instability in memory devices. These results demonstrated that UV–ozone oxidation produced a conformal and well-controlled oxide shell with an atomically sharp interface.

S5. Oxide Thickness Measurement

The thickness of the BOS oxide, acting as the tunneling layer in an NVM, critically determines carrier tunneling probability and thus directly impacts memory performance. To quantify oxide growth under specific oxidation parameters, a free-standing s-BOS was first oxidized under defined conditions.

After oxidation, the sample was transferred onto a SiO_2 substrate, and a positive photoresist (S1813) was spin-coated and patterned to define the etching area on the BOS. Buffered oxide etchant (BOE, 0.7%) was then used to selectively remove the oxide at a rate of ~4 nm/s, exploiting the high etch selectivity between BOS oxide and s-BOS. The step height between the etched and unetched regions was measured using atomic force microscopy (AFM) to determine the oxide thickness (Figure S5). AFM measurements were performed in tapping mode at a scan rate of 0.999 Hz, with system noise levels below 0.15 nm in XY and below 0.35 Å in Z, ensuring high accuracy in thickness determination.

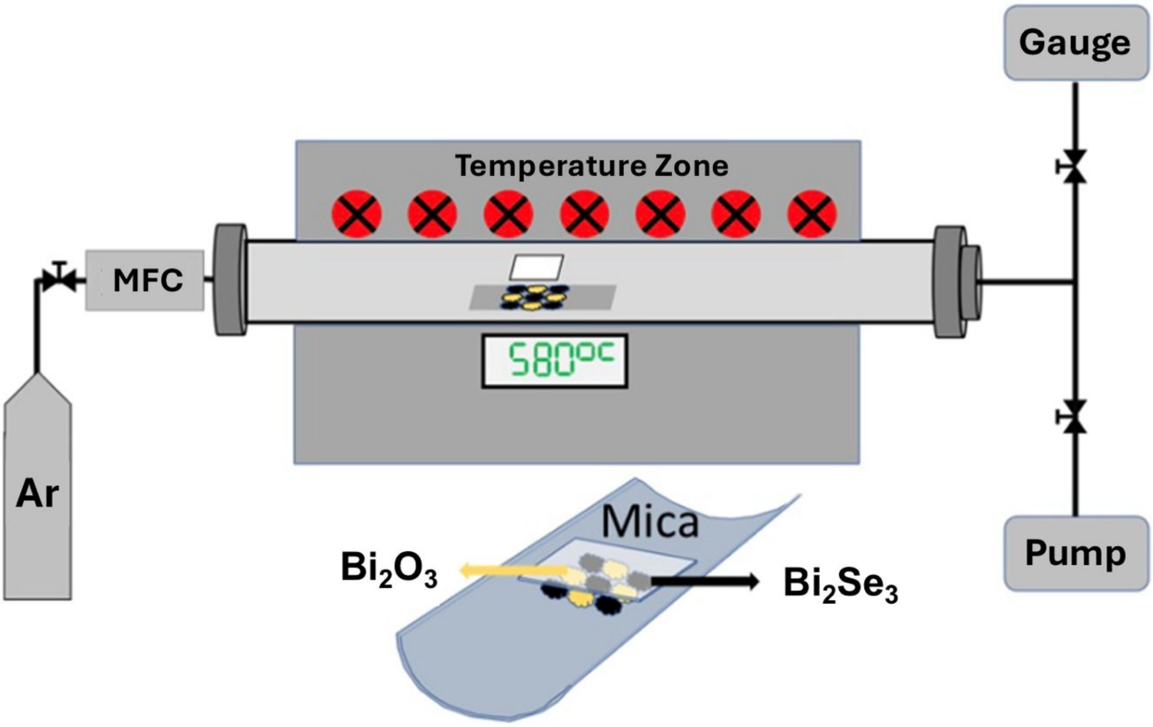

Figure S1 | Schematic illustration of the LPCVD system used for the growth of free-standing Bi_2O_2Se crystals. A 1-inch horizontal quartz tube furnace was used with Bi_2Se_3 and Bi_2O_3 powders arranged in a chessboard pattern as precursors in a quartz boat. The mica substrate was placed face-down above the precursors to promote uniform vapor deposition, with Ar flow as the carrier gas during growth.

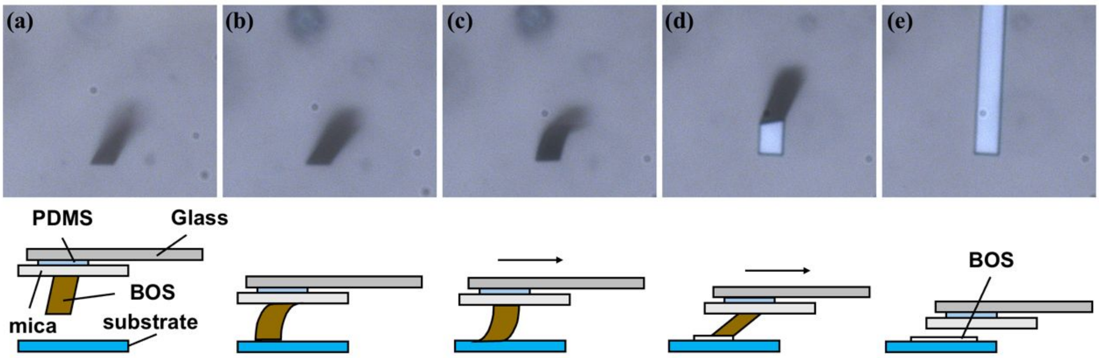

Figure S2 │ Dry transfer of free-standing BOS with OM image. (a-c) The optical image of free-standing BOS crystal, which was the dark crystal with shadow in the middle. When the mica became closer to the target substrate, the end side of the BOS became clear and attracted by the substrate. (d) Since the crystal started bending, we dragged the slide back to make crystal contact to substrate. (e) The crystal completely adhered to the substrate.

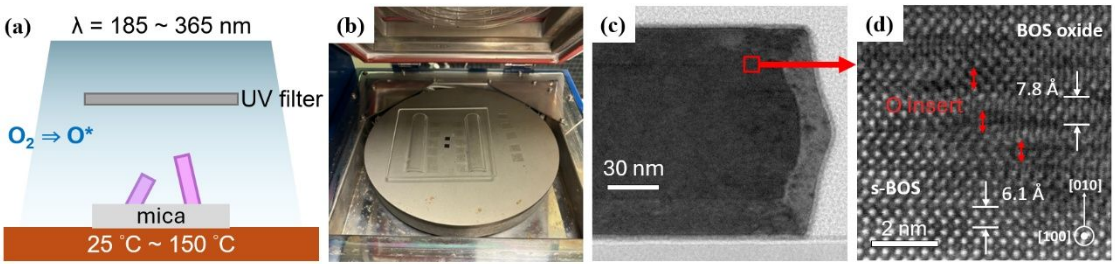

Figure S3 | The Optical and STEM image with schematic diagram for UV–ozone oxidation. (a) Schematic diagram of UV–ozone oxidation treatment to the free-standing BOS crystals on the mica. (b) SAMCO UV-1 system. (c) STEM image showing the s-BOS core with 30 nm BOS oxide shell. (d) By zooming in on the interface of s-BOS/oxide, you can see the region where O is inserted into the Se layer to form β-Bi_2SeO_5.

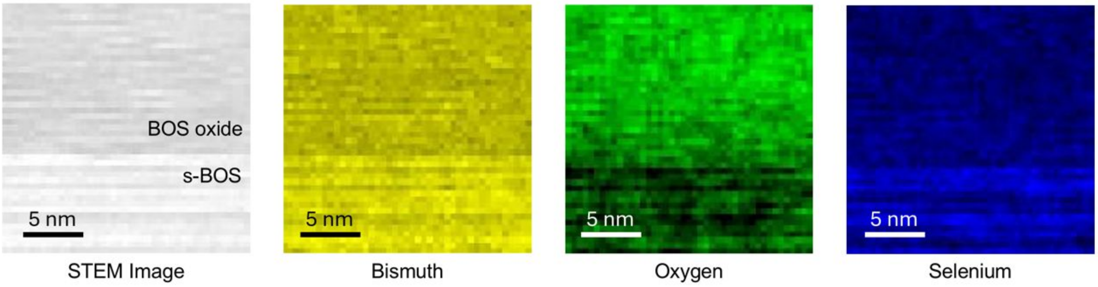

Figure S4 | STEM image and corresponding EELS elemental mappings of the s-BOS/oxide heterostructure. The bright-field STEM image (left) reveals a clear bilayer structure with a s-BOS and an upper BOS oxide. Elemental maps show the distribution of Bi (yellow), O (green), and Se (blue). The oxygen signal is especially concentrated in the shell region with a sharp interface, confirming the formation of a conformal oxide shell with a well-defined interface.

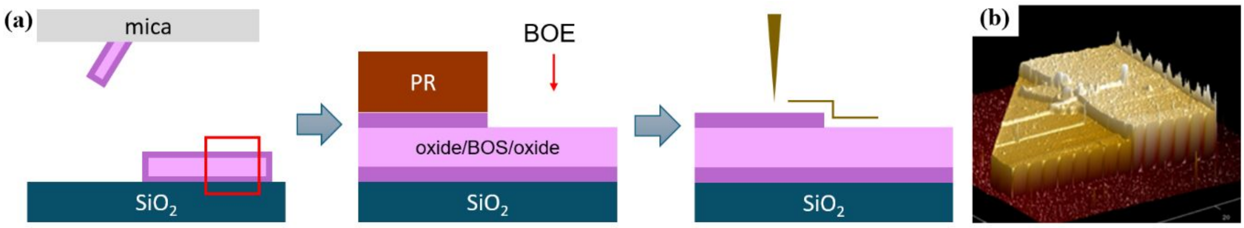

Figure S5 | The measurement of BOS oxide thickness. (a) Utilizing the high etch selectivity of BOE etchant between BOS oxide and m-BOS, the former can be selectively etched in the patterned regions. The thickness of BOS oxide can then be determined by measuring the step height at the interface using AFM. (b) The 3D AFM measurement of the etching step.
